# Comprehensive evaluation of global health cities development levels

**DOI:** 10.3389/fpubh.2024.1437647

**Published:** 2024-07-18

**Authors:** Yu Wen, Yulan Li, Yan Zhang, Bingbing Liu

**Affiliations:** ^1^School of Arts and Design, Yanshan University, Qinhuangdao, China; ^2^Department of Humanities and Social Sciences, Hebei University of Environmental Engineering, Qinhuangdao, China

**Keywords:** health city, evaluation system, entropy weight-TOPSIS, rank-sum ratio (RSR), global health

## Abstract

**Introduction:**

How to scientifically assess the health status of cities and effectively assist in formulating policies and planning for health city development remains a profound challenge in building a global “health community.”

**Methods:**

This study employs the Building Research Establishment’s International Healthy Cities Index (BRE HCI), encompassing ten environmental categories and fifty-eight indicators, to guide and support the scientific development of healthy cities. The entropy weight-TOPSIS method and the rank sum ratio (RSR) method were applied to comprehensively rank and categorize the health development levels of fifteen global cities. Furthermore, through cluster analysis, this research identifies universal and unique indicators that influence the development of healthy cities.

**Results:**

The results indicate that: (1) Within the scope of 58 evaluation indicators, the precedence in weight allocation is accorded to the kilometres of bicycle paths and lanes per 100,000 population (0.068), succeeded by m2 of public indoor recreation space *per capita* (0.047), and kilometres of bicycle paths and lanes per 100,000 population (0.042). (2) Among the ten environmental categories, the top three in terms of weight ranking are transport (0.239), leisure and recreation (0.172), and resilience (0.125). Significant disparities exist between different cities and environmental categories, with the issue of uneven health development within cities being particularly prominent. (3) The study categorizes the development levels of healthy cities into three tiers based on composite scores: it classifies Singapore, Shanghai, and Amsterdam at an excellent level; places Dubai and Johannesburg at a comparatively poor level; and situates the remaining ten cities at a moderate level. (4) The analysis identifies 53 international common indicators and 5 characteristic indicators from the 58 indicators based on the significance of the clustering analysis (*p* < 0.05).

**Discussion:**

The study proposes four strategic recommendations based on these findings: establishing a comprehensive policy assurance system, refining urban spatial planning, expanding avenues for multi-party participation, and augmenting distinctive health indicators. These measures aim to narrow the developmental disparities between cities and contribute to healthy global cities’ balanced and sustainable growth. However, due to existing limitations in sample selection, research methodology application, and the control of potential confounding variables, further in-depth studies are required in the future.

## 1 Introduction

The rapid development of urbanization globally has significantly improved residents’ material standard of living and quality of life. However, it has also presented unprecedented threats and challenges to public health and safety. These challenges include environmental pollution caused by industrial production, motorized transportation, and the spread of diseases triggered by population concentration and mobility, among other urban issues (1). According to the United Nations “World Population Prospects 2022,” the global urban population is expected to grow from 3.3 billion in 2007 to 6.3 billion by 2050 (2). This exponential population growth sharpens the contradiction between urban development and human health. In this context, the construction of healthy cities, as an effective strategy for managing “urban diseases,” has garnered widespread attention worldwide (3). To effectively address global health challenges, it is crucial to accurately understand the trends in urban health development worldwide, evaluate urban health status, identify health issues and weaknesses, design and implement targeted measures, and monitor and provide feedback on their effectiveness. This comprehensive process constitutes the evaluation framework for healthy cities and is an essential policy tool. As a tool for comprehensively measuring the health levels of urban residents and evaluating the factors influencing urban environmental health, the healthy city assessment index system plays a leading and guiding role in meeting the urgent need for continuous improvement and promoting high-quality development of healthy cities.

“Healthy City” originated from the survey Report on the Health status of the British Working Population published by the British scholar Edwin Chadwick in 1842, followed by the establishment of the British Healthy Cities Association (4). In 1984, the World Health Organization (WHO) first proposed the concept of a “healthy city” at the “Health Toronto 2000” conference in Canada, calling for extensive cooperation between departments, institutions and the public to address health cities and health-related issues (5). In 1986, the WHO launched the Healthy Cities Project (6), which aims to improve public health and public health through urban planning. In 1994, WHO defined a healthy city as “a healthy city that continuously develops, develops the natural and social environment, and expands social resources so that people can support each other in enjoying life and exerting their full potential” (7). Since then, the global concept has carried out research on health-supportive environment building (8), health impact assessment (9), health cities planning (10), developed health cities plans and launched a health cities campaign. So far, the campaign has covered (11) in 7,500 cities worldwide.

Developing an evaluation system for healthy cities, based on the concept of “Healthy City, “is an indispensable policy tool to guide the construction of healthy cities (12). By utilizing indicators to measure the health status of cities, the system provides a deeper understanding of the level of health of city development. It reveals the gap with the ultimate goals of “Healthy Cities.” As such, it plays a crucial role in guiding the construction of healthy cities and acting as a benchmark for progress (13). The WHO European Healthy Cities Network introduced 53 Healthy Cities Indicators (HCIs) in 1992, which marked the first systematic collection and analysis of health city data in Europe (14). Further refinement, building on this initial analysis, identified core indicators that are more closely and effectively related to healthy cities, thus optimizing the list to 32 secondary indicators (15). These indicators include qualitative and quantitative data to facilitate comprehensive and feasible data collection. Based on this framework, scholars have developed indicator systems focused on sustainable development (16), eco-cities (17), low-carbon cities (18), and smart cities (19) to measure the sustainable development and ecological livability of healthy cities. However, these indicators primarily focus on sustainable development capabilities and the ecological environment, with less attention given to cities’ overall health development status.

Current evaluative research on the ‘healthy cities’ developmental level predominantly focuses on three aspects. Firstly, grounded in public health ideology, emphasis is placed on the public health status at national or regional levels. The World Health Organization 2015 published the “Global Reference List of 100 Core Health Indicators” (20), which organizes these indicators into four analytical categories: inputs and processes, outputs, outcomes, and impacts. This framework enables stakeholders to comprehend the interconnectivity among indicators and their respective roles in the regulatory mechanisms of health city systems, thus facilitating the formulation of more effective intervention strategies. In response to the State Council of China’s directive for “establishing a health cities construction indicator and evaluation system suited to our national conditions,” as stated in the “Opinions on Further Strengthening Patriotic Health Work in the New Era” from 2018, the Chinese Patriotic Health Campaign Committee devised an indicator system for healthful urban environments that reflects China’s unique socio-economic context (21). Secondly, based on the concept of significant health, the index setting is no longer limited to the field of public health; it pays attention to the three dimensions of various health-influencing factors, health services, and health status. For example, Remington P L et al. established the US County Health Ranking Index (County Health Rankings, CHR) based on health outcomes and factors to quantify the health differences (22) between counties and counties in the United States. Barboza et al. quantitatively assesses the health effects (23) of different types of green space exposure in 980 cities in 31 European countries. Third, a comprehensive set of indicators is employed to form a holistic assessment based on the broad concepts of health and sustainable development, encompassing multiple dimensions such as socio-economics, industrial employment, education, culture, and public safety. For example, Lv et al. (24) used the full-array polygon (EAP) method to evaluate and compare the environment, economy, population, service, space and overall health status of the typical cities in China. Yan et al. (25) analyzed the health status of 258 cities in China, using the perspective of a healthy environment, healthy society, health services, healthy population, healthy economy and healthy culture. Pineo et al. (26) adopted the Delphi expert consultation method, cooperation with pilot cities, and continuous improvement to establish the international Healthy Cities Index evaluation system (BRE HCI). They evaluated the healthy development of different global cities through two weight systems to provide a scientific basis for formulating more perfect health city planning policies.

Existing literature indicates that current research on the evaluation of healthy cities is predominantly limited to single countries, individual cities, and specific urban clusters, lacking comparative analysis across cities from different countries globally. Additionally, in terms of evaluation methodologies, existing studies either entirely rely on quantified statistical data or employ expert consultation to select evaluation systems, which are then combined with methods such as the Analytic Hierarchy Process (AHP), Entropy Weight method, and TOPSIS method to determine indicator weights and calculate comprehensive level values. However, these studies often depend on a singular evaluation method, which may not accurately or comprehensively reflect the developmental levels of healthy cities. Therefore, we recommend fully leveraging the complementary advantages of various evaluation methods to conduct a more comprehensive assessment (27). By integrating the Entropy Weight Method with the TOPSIS method, it is possible to eliminate the effects of differing units of measurement, and the approach does not impose specific restrictions on sample size or data distribution. However, this combined method does not categorize or tier the sample results. The rank sum ratio (RSR) method can address the categorization and tiering that the Entropy-TOPSIS method does not fulfill. Nevertheless, due to its non-parametric transformation, the RSR method can result in the loss of original data, a deficiency that the Entropy-TOPSIS method can rectify. The synergistic application of the Entropy-TOPSIS and RSR methods can overcome the limitations inherent in single evaluation approaches, rendering the assessment results more objective and applicable.

The study is based on 707 cities that were part of the Globalization and World Cities Research Network (GaWC) ranking in 2020. It considers the characteristics of the research area’s high integration with the global economy and the availability of indicator data to select 15 cities as the research subjects. The entropy weight-TOPSIS and rank sum ratio methods are comprehensively applied to evaluate these cities’ health development levels. The aim was to identify and analyze the differences in health development levels among global cities, assist urban management departments in accurately pinpointing strengths and issues, and reasonably assess and diagnose the overall health levels and deficiencies of the sample cities. Further, cluster analysis identifies common and unique indicators for evaluating cities’ health across different countries to implement city-specific policies. This approach provides pathways and scientific strategies for healthy city planning, optimizes the healthy city development goals, makes up for the shortcomings of healthy city construction, and tackles prominent issues of uneven health city development, which is conducive to the realization of the goal of promoting sustainable development of living environment.

## 2 Materials and methods

### 2.1 Study area and indicators selection

#### 2.1.1 Study area

**Figure 1:**
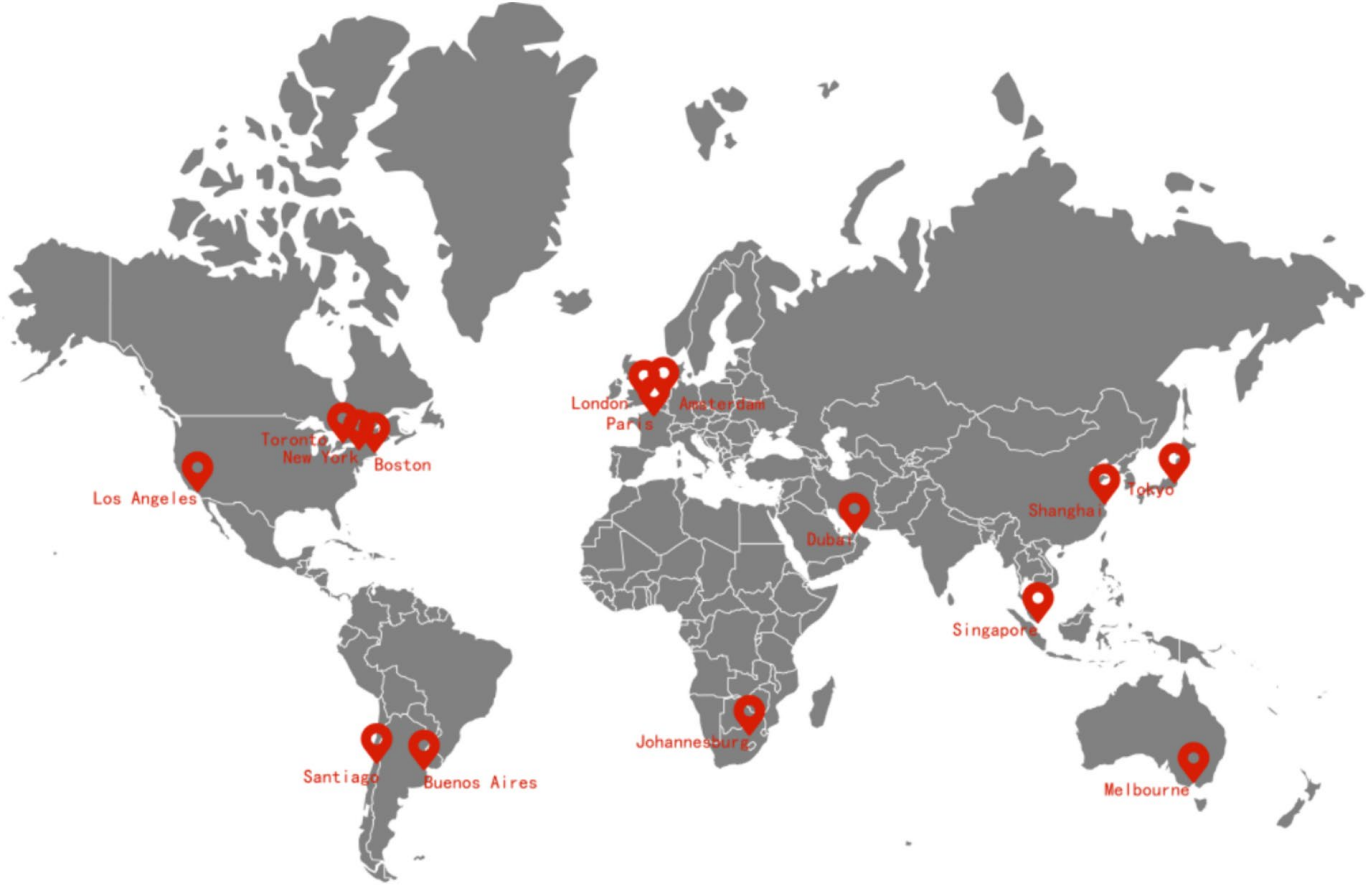
Schematic illustration of the study areas’ geographical locations.

Based on the city classification standards developed by the Globalization and World Cities Research Network (GaWC), major cities worldwide are categorized into Alpha, Beta, and Gamma levels according to their connectivity with the global economy (28). Alpha-level cities are major metropolises that play a leading role in global political, economic, and other social activities and can influence and drive development. A city’s economic advantages can create a siphon effect, attracting a large influx of high-quality talent (29) and capital (30), making it easier for these cities to finance, construct, and maintain public infrastructure (31), offering higher-quality health services, social support, and welfare programs. Such developments can contribute to reducing social inequalities and enhancing the overall health literacy of residents. Additionally, Alpha-level cities often serve as pioneers in constructing healthy cities, and their successful experiences and strategies can offer valuable references and lessons for other cities. Thus, considering the potential impact of economic development levels on the construction of healthy cities and data availability, 15 Alpha-level cities closely linked to the world’s major economic regions with significant economic growth were selected as research cases. These cities include Amsterdam, Boston, Buenos Aires, Dubai, Johannesburg, London, Los Angeles, Melbourne, New York, Paris, Santiago, Shanghai, Singapore, Tokyo, and Toronto. Their geographic locations are illustrated in Figure 1.

#### 2.1.2 Selection of the evaluation system

The research adheres to scientific rigor, systematic analysis, and operational feasibility principles. It utilizes the evaluation indicator system of 10 environmental categories and 58 indicators from the Building Research Establishment’s International Healthy Cities Index (BRE HCI). This index offers a comprehensive assessment framework covering multiple dimensions, including healthcare, environmental quality, social culture, and economic development, ensuring a holistic evaluation approach (26). The BRE HCI has garnered widespread international recognition. Its methodology, based on extensive, objective data, ensures the scientific accuracy and comparability of evaluations among different cities’ health development levels. Moreover, the continuous updating and dynamics of its data can help research capture and analyze the latest trends in healthy urban development and provide adequate decision support for policymakers. Therefore, using the BRE International Healthy Cities Index as the evaluation indicator system in this study not only promotes in-depth theoretical and practical exploration but also enhances the applicability of the research findings. Furthermore, this evaluative schema has been independently applied to the mentioned 11 cities, indicating that the screening of these cities has good applicability.

#### 2.1.3 Data sources

The evaluation indicator data is sourced from numerous international open data repositories, including the World Bank Open Data (WBOD), UN-Habitat’s Global Urban Observatory, the World Council on City Data (WCCD), the World Health Organization (WHO), as well as official statistical data websites of various countries. To address gaps in the data, a scientific quantitative analysis approach is employed in which the average annual growth rate is calculated using data from adjacent years to fill in missing values. In cases where individual indicator gaps are too large to be filled using the above method, strictly adhering to relevant ethical norms and legal regulations is essential. Using the Chrome browser’s Developer Tools, perform a detailed analysis of official websites or public data platforms (such as government transportation departments, environmental agencies, statistics bureaus, and other relevant authorities) that publish relevant indicator data. Locate and obtain the request data packets that contain the list of issues and parse the JSON data to extract all question codes. Subsequently, write a Python web scraping program to obtain and process data corresponding to the relevant year’s keywords for the indicators. Finally, the panda’s library will store the extracted data into a CSV file for subsequent analysis and processing, ensuring the data’s legality, continuity, and completeness. Ultimately, the study collected and organized 870 data points across 58 indicators, as detailed in Table 1.

**Table 1:** Sources of indicator data.

Category	Identifier	Indicator	Data source
Air quality	A1	Concentration of PM2.5 μg/m^3^	World Bank Open Data (2020)
	A2	Concentration of NO2 μg/m^3^	World Bank Open Data (2020)
	A3	Concentration of PM10 μg/m^3^	World Bank Open Data (2020)
	A4	Percentage of population living within 500 m of highway or major road	Urban Planning Agencies of Local Governments
	A5	% of urban land area covered by trees	United Nations Environment Program.
Food access	F1	Signee to Milan Urban Food Policy Pact	Milan Urban Food Policy Pact committee
	F2	Accessibility: Average walking time to food stores selling fruits and vegetables	UN-Habitat
	F3	the % of no vehicle households living beyond 0.9 mile radial distance of a supermarket	Municipal Planning Departments and Housing Agencies.
	F4	Availability (home): Number of food stores selling fruits and vegetables within 500 m of domestic properties.	Local Health or Food Safety Agencies
	F5	Availability (work): Number of food stores selling fruits and vegetables within 500 m of retail and commercial properties.	Local Health or Food Safety Agencies
	F6	% of residents who respond a large selection of low-fat food is available in my neighborhood	Food and Agriculture Organization of the United Nations
Green infrastructure	G1	Hectares of green area per 100,000 population	Wikipedia
	G2	Percentage change in number of native species	Our World in Data
	G3	% of dwellings < 300 m from green space (min. Size 1 hectare)	Land Survey and Statistics Office
Housing and buildings	H1	Number of homeless people per 100,000 population	Our World in Data
	H2	Number of new building stock certified with a sustainable building standard (e.g., BREEAM and LEED)	BREEAM, LEED and other sustainable building certification bodies
	H3	Housing affordability	Numbeo
	H4	Building Quality Control Index	Numbeo
	H5	% of households living in fuel poverty	Index Mundi
	H6	% of refurbished building stock certified with a sustainable building standard (e.g., BREEAM and LEED)	BREEAM, LEED and other sustainable building certification bodies
Leisure and recreation	L1	m^2^ of public indoor recreation space *per capita*	National Bureau of Statistics
	L2	m^2^ of public outdoor recreation space *per capita*	National Bureau of Statistics
	L3	Access to leisure and recreation facilities: Facilities within <600 m per 100,000	National Bureau of Statistics
	L4	Pre-school playground equipment and physical structures	Local Government and Urban Planning Agencies
	L5	Fitness centers per 100,000 population	Sixth National Sports Ground Survey
	L6	Sports facilities per 100,000 population	Sixth National Sports Ground Survey
Noise pollution	N1	% of population exposed to noise pollution measured at Lden >55 dB	UNEP—UN Environment Programme
	N2	% of residents exposed to noise levels higher than 35 dB during the night	UNEP—UN Environment Programme
	N3	% of residents exposed to noise levels higher than 45 dB during the day	UNEP—UN Environment Programme
	N4	% of residents who report noise annoyance	UNEP—UN Environment Programme
Resilience	R1	Greenhouse gas emissions (tonnes *per capita*)	US Energy Information Administration
	R2	Residential electricity use *per capita* (kWh/year/capita)	US Energy Information Administration
	R3	Electricity consumption of public building per year (kWh/M2)	US Energy Information Administration
	R4	Percentage of energy derived from renewables	US Energy Information Administration
	R5	% of energy efficient building stock	US Energy Information Administration
	R6	Urban heat index	Moderate Resolution Imaging Spectroradiometer, et al.
	R7	% of land area with green space	Department of Urban Planning
	R8	% of land area with reflexive surfaces	World Council on City Data
	R9	% of buildings with green roofs	World Council on City Data
Safety and security	S1	Crimes against property per 100,000 population	UK Home Office’s Recorded Crime Statistics
	S2	% of residents who report presence of environmental cues of crime and vandalism	Statistics Canada. Police Administration Survey 2020
	S3	% of residential neighborhoods with adequate street lighting	Statistics Canada. Police Administration Survey 2020
Transport	T1	% of commuters using a travel mode to work other than a personal vehicle	CEIC Data Global Database
	T2	Kilometers of bicycle paths and lanes per 100,000 population	Index Mundi
	T3	Kilometers of high capacity public transport system per 100,000 people	Local/regional transit authorities2020
	T4	Kilometers of light passenger public transport system per 100,000 people	Local/regional transit authorities2020
	T5	Population density (population per km2)	Open Spatial Demographic Data and Research
	T6	% of roads with speed limits at 20mph or less	OpenStreetMap
	T7	% of residences within 200 m of roads with ≥300 vehicles per hour	OpenStreetMap
	T8	% of roads with sidewalks	OpenStreetMap
	T9	Annual Hours of Delay per Commuter	INRIX 2020 Global Traffic Scorecard
	T10	Street connectivity: average number of three or more-way intersections per square kilometer	OpenStreetMap, GIS, Copernicus Sentinel satellites, et al.
	T11	Land use mix: average number of land use types per neighborhood	GIS, MODIS (2020), the Landsat series of satellites in the US, et al.
Utilities and services	U1	% of population with potable water supply service	Statistics W.H. World Health Organization
	U2	% of population with authorized electricity service	US Energy Information Administration
	U3	% of population served by wastewater collection	Statistics W.H. World Health Organization
	U4	% of population living in slums	Statistics W.H. World Health Organization
	U5	% of population with regular solid waste collection	the Local Operator(s) of solid waste collection systems, census data, and municipal waste facilities.

### 2.2 Research method

The study employs the entropy weight method to determine the objective weights of each indicator. Subsequently, the TOPSIS method is used to compare the relative closeness of the 58 evaluation indicators of the 15 cities to the ideal health cities, thereby determining each city’s overall ranking. Finally, the rank sum ratio method is utilized to sort the results and categorize them into levels of excellence, medium, and poor, providing an intuitive display of the global cities’ health hierarchy levels. This approach facilitates a more in-depth assessment and analysis of the health development levels of the 15 cities.

#### 2.2.1 Entropy weight

The Entropy Weight Method (EWM) represents an objective weighting technique that capitalizes on the inherent properties of the raw data to quantify information content (32). Predicated on the principles of information entropy, this methodology asserts that a reduction in entropy within a system concomitantly increases data dispersion and informational richness, thereby augmenting the associated weights. In contrast, diminished variance in data leads to a reduction in both information content and respective weights. The advantage of the entropy weight method lies in its ability to objectively assign weights based on existing data information, thereby avoiding biases introduced by subjective weighting. This method enhances the objectivity and accuracy of decision-making, offering better precision than subjective methods like the Analytic Hierarchy Process (AHP) (33). The specific steps are as follows:

1. Normalization of Indicators

To eliminate the dimensional and unit differences between the indicators, the data of each index were normalized. Due to the different meanings of positive indicators and negative indicators, the specific calculation formulas are also different, as follows:

Positive indicators—where a higher score reflects a higher level of health cities—the normalization approach is as Eq. 1:

(1) $$y_{ij}=\frac{x_{ij}-\min\left(x_{j}\right)}{\max\left(x_{j}\right)-\min\left(x_{j}\right)}$$

Negative indicators—where a higher score indicates a lower level of health cities—the normalization approach is as Eq. 2:

(2) $$y_{ij}=\frac{\max\left(x_{j}\right)-x_{ij}}{\max\left(x_{j}\right)-\min\left(x_{j}\right)}$$

Where $y_{ij}$ represents the normalized datum of the j evaluative criterion corresponding to the i specimen, where i ranges from 1 to m , m being the cumulative count of specimens, and j spans from 1 to n , n signifying the total indicators within the analyzed system. Herein, $y_{ij}$ specifies the raw score of the i specimen’s j criterion. The terms $\max\left(x_{j}\right)$ and $\min\left(x_{j}\right)$ delineate the extremities of the observed spectrum for the j criterion, interpreted as the maximal and minimal valuations, respectively.

1. Calculate the specific gravity $P_{ij}$ of the j index under the i samples Eq. 3:

(3) $$P_{ij}=\frac{y_{ij}}{\sum_{i=1}^{m}y_{ij}}$$

1. Calculation of the Information Entropy $e_{j}$ for the j Indicator Eq. 4:

(4) $$e_{j}=-\frac{1}{\ln_{n}}\overset{n}{\underset{i=1}{\sum }}P_{ij}\ln P_{ij}\;\text{,}k=\frac{1}{\ln_{n}}>0,e_{j}\geq 0$$

1. Calculating the Weight of the j Indicator Eq. 5:

(5) $$w_{j}=\frac{1-e_{j}}{\sum_{i=1}^{m}\left(1-e_{j}\right)}$$

#### 2.2.2 TOPSIS

The Technique for Order of Preference by Similarity to Ideal Solution (TOPSIS), proposed by C.L. Hwang and K. Yoon in 1981, is a multi-criteria decision analysis method. Its principle lies in selecting the option closest to the positive ideal solution and furthest from the negative one. A comprehensive evaluation index is derived by calculating the degree of closeness and distance of the evaluated object to the ideal solution (34), accurately reflecting the disparities among the evaluated targets (35). The computational steps are as follows:

1. Constructing the Weighted Normalization Matrix R Eq. 6:

(6) $$R=w_{j}*z_{ij}=\left[\begin{array}{cccc} w_{1}*z_{11} & w_{2}*z_{12} & \cdots & w_{n}*z_{1n}\\ w_{1}*z_{21} & w_{2}*z_{22} & \cdots & w_{n}*z_{2n}\\ \vdots & \vdots & \cdots & \vdots \\ w_{1}*z_{m1} & w_{2}*z_{m2} & \cdots & w_{n}*z_{mn} \end{array}\right]$$

1. Calculation of the Positive and Negative Ideal Solutions $R^{+}$ Eq. 7, R^–^ Eq. 8:

(7) $$R^{+}=\left\{R_{1}^{+},R_{2}^{+},\cdots ，R_{n}^{+}\right\}=\left\{\max\;R_{ij}|i=1,2,\cdots ,m;j=1,2,\cdots ,n\right\}$$

(8) $$R^{-}=\left\{R_{1}^{-},R_{2}^{-},\cdots ，R_{n}^{-}\right\}=\left\{\max\;R_{ij}|i=1,2,\cdots ,m;j=1,2,\cdots ,n\right\}$$

1. Calculation of the Euclidean Distance from the Evaluated Objects to the Positive and Negative Ideal Solutions $D_{i}^{+}$ Eq. 9, $D_{i}^{-}$ Eq. 10:

(9) $$D_{i}^{+}=\sqrt{\overset{n}{\underset{j=1}{\sum }}{\left(R_{ij}-R_{j}^{+}\right)}^{2}}$$

(10) $$D_{i}^{-}=\sqrt{\overset{n}{\underset{j=1}{\sum }}{\left(R_{ij}-R_{j}^{-}\right)}^{2}}$$

1. Calculation of the Relative Closeness to the Ideal Solution for Each Evaluation Object $C_{i}$ (Composite Scores) Eq. 11:

(11) $$C_{i}=\frac{D_{i}^{-}}{D_{i}^{-}+D_{i}^{-}}$$

Where $C_{i}$ represents the level of a health cities, with values ranging from 0 to 1. A higher value indicates that the healthiness level of the city under study is closer to the optimal level. Drawing upon the classification method used by Lei et al. (36) for assessing land use performance, the natural breaks method (Jenks) is employed to divide the proximity values into four distinct categories. The specifics are illustrated in Table 2.

**Table 2:** Evaluation criteria for the development level of healthy cities.

Proximity value range	Health level
(0.0.3]	Substandard
(0.3, 0.6]	Moderate
(0.6, 0.8]	Good
(0.8, 1]	Excellent

#### 2.2.3 RSR

The Rank-Sum Ratio (RSR) method, proposed by Tian Fengtiao in 1988, is a multi-indicator assessment method based on non-parametric statistics. Its principle is based on converting data into ranks, creating a dimensionless statistical measure called the RSR, which is then used to sort and grade the objects of study (37). The RSR method addresses the limitations of the entropy weight-TOPSIS method, which cannot classify evaluation results. It makes the evaluation process more scientific and practical and the results more objective and reliable. The steps of the RSR method are as follows:

1. Assign Ranks and Calculate the rank-sum ratio:

In conducting the study, the obtained proximity values of each criterion are employed as ranks within the Rank-Sum Ratio (RSR) method. These ranks are then ordered in an ascending fashion, from the smallest to the largest RSR value, to assign the rank order for each group.

1. Calculate the cumulative frequency Eq. 12:

(12) $$P=\bar{R}/n*100\%$$

The cumulative frequency, denoted as P, was converted into a probability measure using the NORMSINV function. Lower resultant values indicated a superior evaluation of the subject under consideration.

1. Calculate the linear regression equation Eq. 13:

Using SPSS 26.0 software to build a regression equation with Probit as independent variable and RSR ( $C_{i}$ ) value as dependent variable, namely:

(13) $$C_{i}=a+b*\mathrm{Probit}$$

1. Classification and ranking:

By checking the regression equation, the RSR critical value is output, which is divided into <4,4~ and 6~three grades according to the unit of probability Probit , which means difference, medium and excellent, and ranks each city.

## 3 Results

### 3.1 Sub-indicator weights results

**Figure 2:**
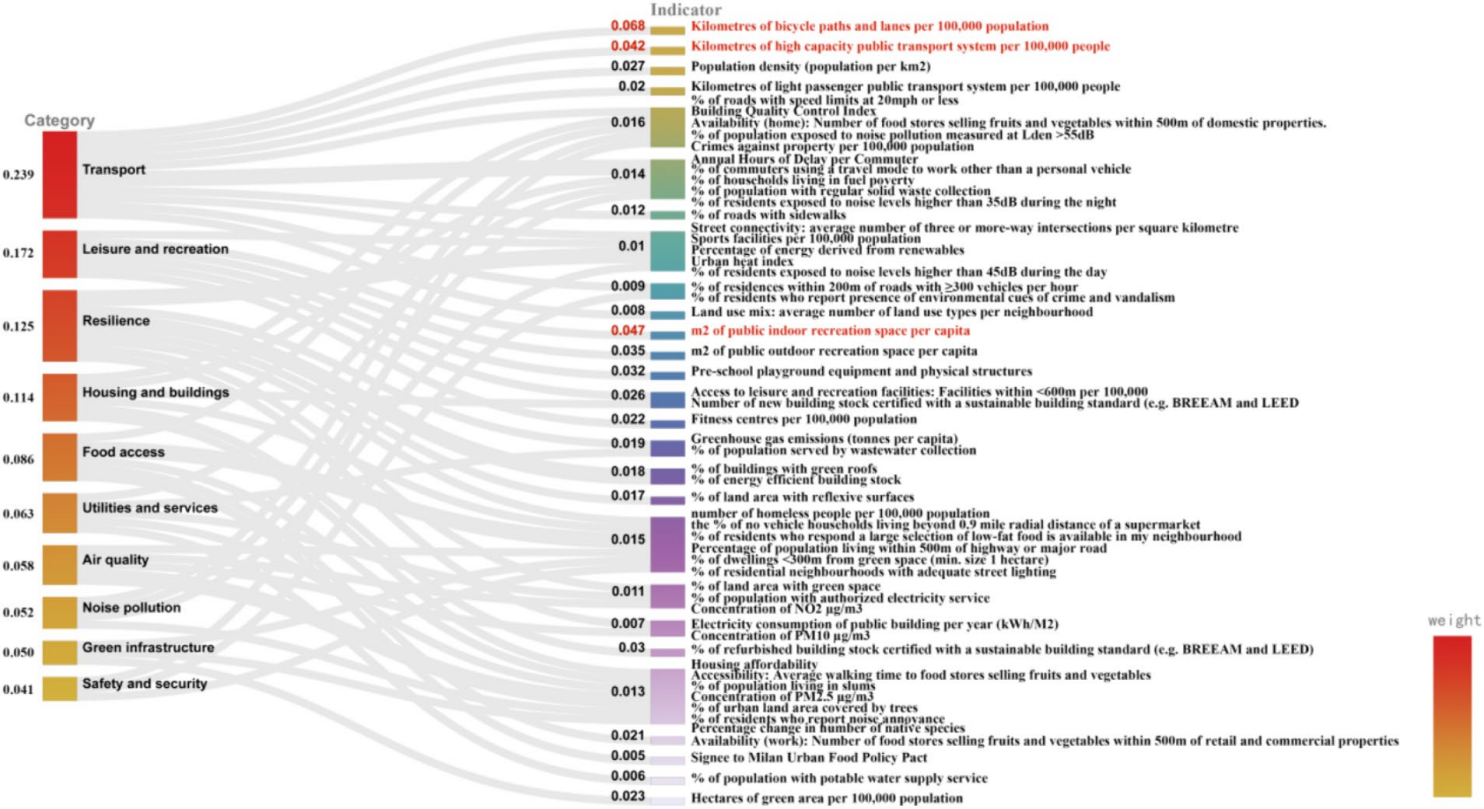
Sub-indicator weights of each environmental category in the healthy cities evaluation system.

The outcomes of the weight calculations for 58 evaluative indicators about the development levels of 15 healthy cities via the Entropy Weight Method are presented in Figure 2. Within these 58 secondary indicators, the top three, arranged by weight, are the kilometers of bicycle paths and lanes per 100,000 population (0.068), m2 of public indoor recreation space *per capita* (0.047), and kilometers of bicycle paths and lanes per 100,000 population (0.042). The indicator attributed with the minimum weight is the endorsement of Signee to Milan Urban Food Policy Pact” (0.005).

In the category of air quality, the indicator with the highest weight is the percentage of population living within 500 m of highway or significant road (0.015); within the category of food access, the highest weighted indicator is availability (work): number of food stores selling fruits and vegetables within 500 m of retail and commercial properties (0.021); in green infrastructure, the most heavily weighted indicator is hectares of green area per 100,000 population (0.023); in the housing and buildings category, the highest weight is given to % of refurbished building stock certified with a sustainable building standard (e.g., BREEAM and LEED) (0.030); for the category of leisure and recreation, the most significant weight is placed on m2 of public indoor recreation space *per capita* (0.047); in the noise pollution category, the most significant weight is assigned to % of population exposed to noise pollution measured at Lden > 55 dB (0.016); in the category of resilience, the weightiest indicator is greenhouse gas emissions (tonnes *per capita*) (0.019); within the safety and security category, the highest weight is attributed to Crimes against property per 100,000 population (0.016); in the transportation category, the highest weighted indicator is the number of kilometers of bicycle paths and lanes per 100,000 population (0.068); and in the category of utilities and services, the highest weight is given to % of population served by wastewater collection (0.019).

Within the global assessment of health city levels across 10 environmental categories, the top three with the highest weights are transport (0.239), leisure and recreation (0.172), and resilience (0.125). This indicates that these three categories play a significant role in affecting the health levels of cities worldwide. Conversely, the three categories with the lowest weights are safety and security (0.041), green infrastructure (0.050), and noise pollution (0.052), suggesting that their impact on the global health of city levels is comparatively weaker.

### 3.2 Environmental category evaluation results

The TOPSIS method calculates the distance of each evaluation indicator to the ideal target (Di+) and the negative-ideal target (Di-), ultimately determining the closeness of the evaluated object to the optimal goal. A Ci value closer to 1 indicates a more favorable evaluation of the object. According to Table 3, within the most influential factor of global health cities levels, the transport category, only 33.33% of cities reach a moderate level, and 60.00% perform poorly. This suggests considerable room for improvement in this dimension among the 15 cities evaluated. Singapore leads with a proximity value of 0.592, indicating its transportation system is the closest to the ideal state among the cities studied; Los Angeles has the lowest proximity value at 0.271, highlighting an urgent need for improvements in its transportation sector. In the leisure and recreation category, Singapore once again leads with the highest proximity value of 0.658, showcasing its superior leisure and recreational facilities compared to the other cities; Buenos Aires ranks last with a proximity value of 0.149, indicating significant potential for development in its leisure and recreation offerings. In the resilience category, Paris has the highest proximity value at 0.752, signifying a reasonable level and indicating that Paris performs best in terms of resilient design; New York has the lowest proximity value at 0.343, which suggests it has considerable scope for improvement in resilience practices compared to other cities. In the category of safety and security, which has a minor impact on global cities’ overall health development levels, Johannesburg has the highest proximity value at 0.644, suggesting that it performs best in safety and security matters. In contrast, Dubai has the lowest proximity value at 0.356, indicating the poorest performance in this category. In the category of green infrastructure, most cities are at a medium level (73.34%), with Boston having the highest proximity value at 0.683. This indicates that Boston’s green infrastructure is the best among the 15 cities studied, whereas Tokyo, with the lowest proximity value at 0.330, has room for improvement. The category of noise pollution showcases particularly notable variations in performance among cities; Shanghai has the highest proximity value at 0.925, demonstrating exceptional control over noise pollution among the cities, while Tokyo’s lowest proximity value at 0.171 suggests that it has a severe noise pollution issue.

**Table 3:** Composite closeness values of each environmental category in the health cities evaluation system.

City	Air quality	Food access	Green infrastructure	Housing and buildings	Leisure and recreation	Noise pollution	Resilience	Safety and security	Transport	Utilities and services
Category										
Amsterdam	0.738	0.538	0.631	0.420	0.430	0.429	0.643	0.426	0.498	0.764
Boston	0.713	0.555	0.683	0.280	0.329	0.528	0.563	0.442	0.355	0.574
Buenos Aires	0.766	0.428	0.625	0.464	0.149	0.515	0.601	0.629	0.364	0.506
Dubai	0.224	0.397	0.376	0.445	0.281	0.682	0.485	0.356	0.278	0.486
Johannesburg	0.349	0.484	0.589	0.364	0.144	0.536	0.655	0.644	0.254	0.397
London	0.623	0.474	0.442	0.306	0.484	0.671	0.608	0.546	0.458	0.548
Los Angeles	0.483	0.581	0.361	0.613	0.403	0.353	0.573	0.516	0.271	0.449
Melbourne	0.749	0.285	0.488	0.542	0.370	0.592	0.355	0.552	0.427	0.674
New York	0.608	0.807	0.636	0.231	0.642	0.592	0.343	0.498	0.460	0.565
Paris	0.624	0.628	0.331	0.422	0.485	0.487	0.752	0.569	0.433	0.619
Santiago	0.700	0.453	0.327	0.486	0.230	0.705	0.514	0.600	0.308	0.680
Shanghai	0.395	0.671	0.548	0.648	0.528	0.925	0.486	0.436	0.478	0.660
Singapore	0.746	0.495	0.352	0.653	0.658	0.471	0.407	0.392	0.592	0.607
Tokyo	0.747	0.687	0.330	0.630	0.514	0.171	0.718	0.368	0.368	0.513
Toronto	0.768	0.581	0.441	0.547	0.454	0.668	0.348	0.457	0.342	0.632

### 3.3 Composite evaluation results of healthy cities development levels

**Figure 3:**
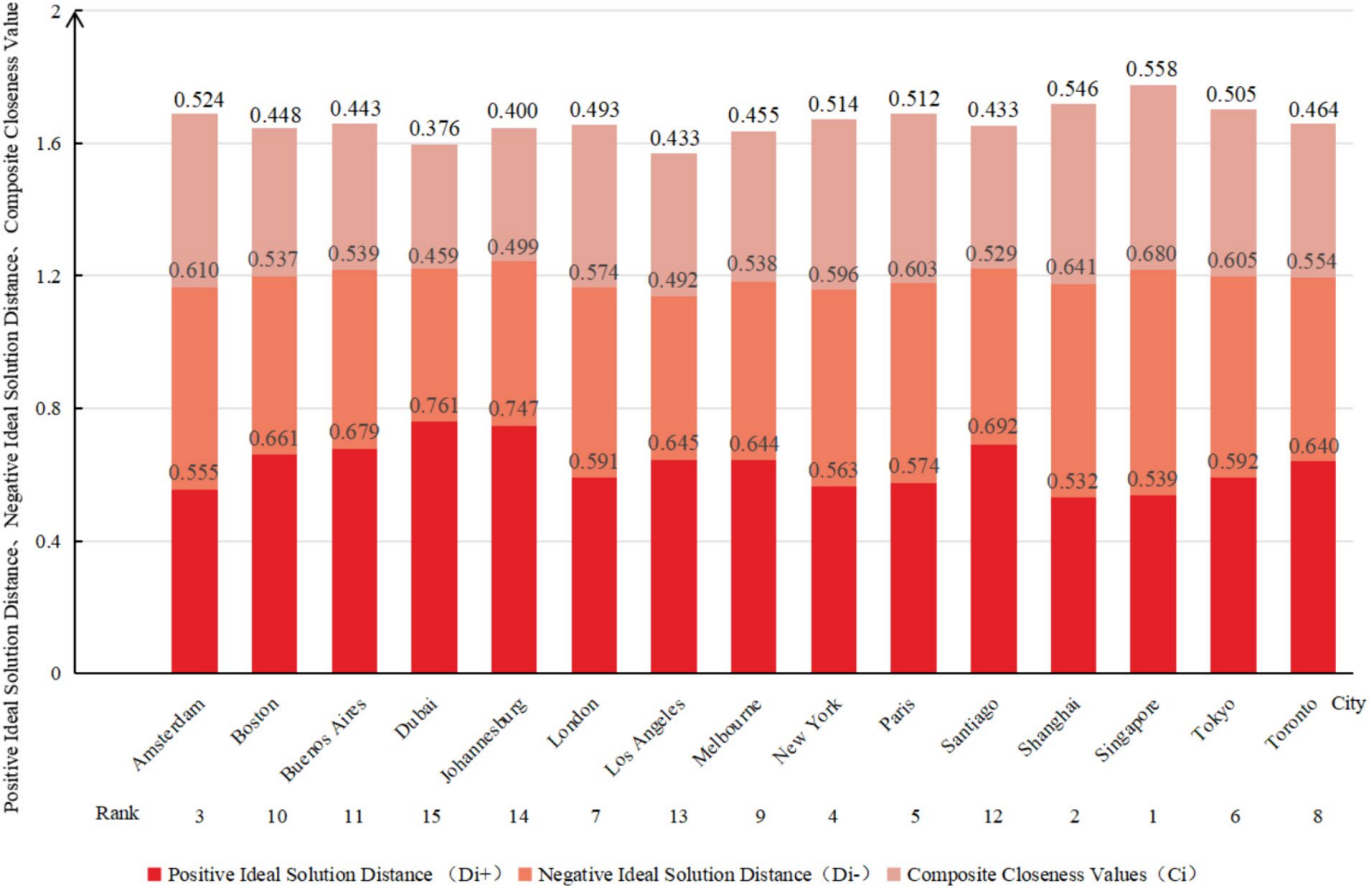
Composite closeness values and rankings of 15 healthy cities.

The composite evaluation result is based on the closeness-to-ideal scores across 10 environmental categories, including air quality, food accessibility, green infrastructure, etc. The TOPSIS method is utilized to calculate the urban health scores, which determine the cities’ ranking. This method provides a comprehensive measure of the level of urban health development. From the composite closeness-to-ideal scores and rankings of the 15 global cities in Figure 3, the top three cities are Singapore, Shanghai, and Amsterdam, indicating superior overall health levels. Conversely, Dubai, Johannesburg, and Los Angeles are the bottom three cities.

**Figure 4:**
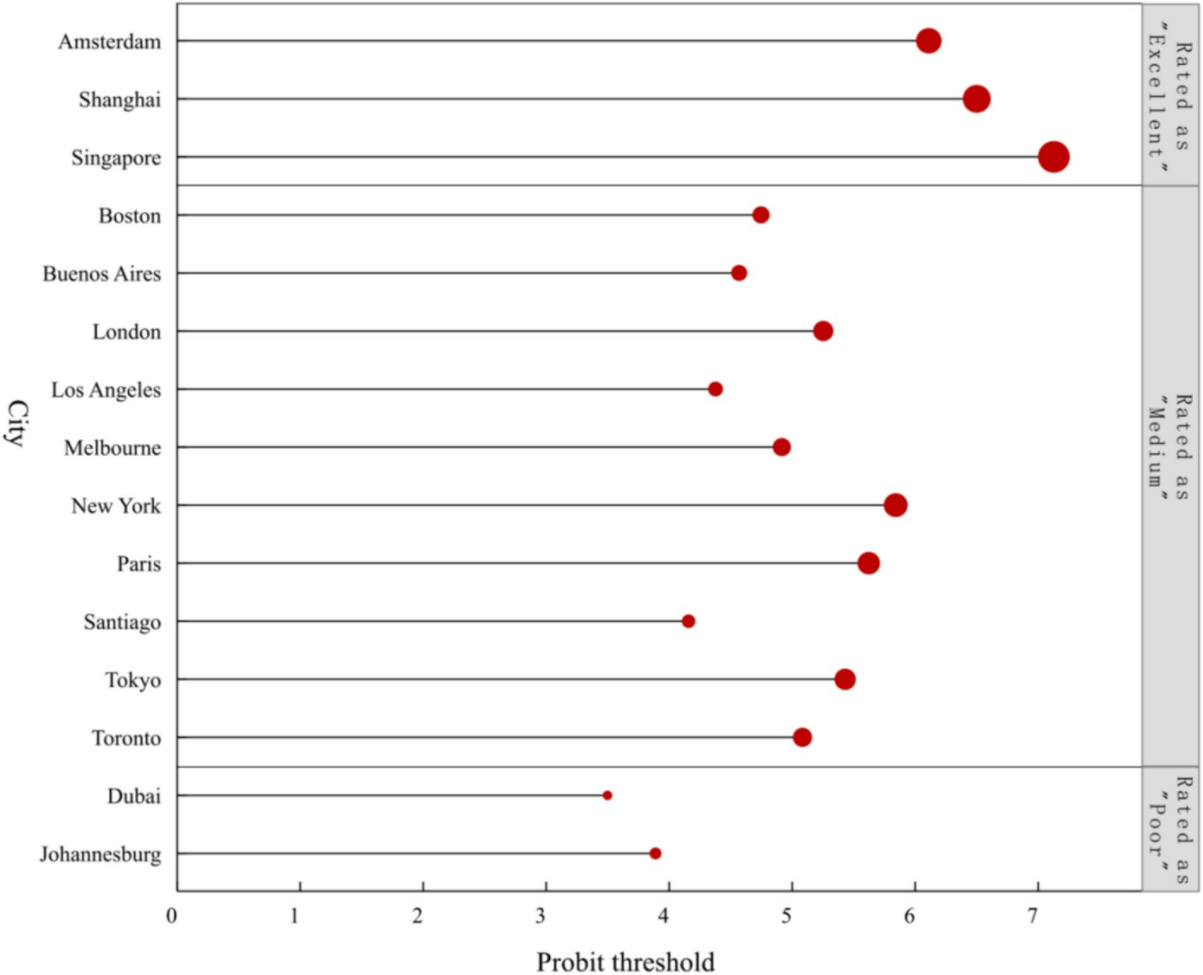
Stratification results for the health development levels of 15 cities.

Applying Probit and RSR values presented in Table 4, a regression equation was formulated (*F* = 316.304, *p* < 0.001) with a coefficient of determination R^2^ of 0.961, substantiating the statistical significance of the regression model. The study conformed to the RSR optimal stratification criteria (38), stipulating that each division should encompass at least two cases. According to the reasonable grading table, the health levels of the 15 cities were segregated into three tiers: poor, medium, and excellent. Dubai and Johannesburg are classified as “poor” (13.3%), while Singapore, Shanghai, and Amsterdam are classified as “excellent” (20.0%). The remaining cities are categorized as “medium” (66.7%). Detailed stratification results are illustrated in Figure 4.

**Table 4:** Distribution results according to the RSR method.

City	RSR ranking	Probit threshold	RSR fitted value	RSR threshold
Amsterdam	3	6.111	0.566	>0.558
Shanghai	2	6.501	0.594	
Singapore	1	7.128	0.639	
Boston	10	4.747	0.469	0.415–0.558
Buenos Aires	11	4.569	0.456	
London	7	5.253	0.505	
Los Angeles	12	4.377	0.442	
Melbourne	9	4.916	0.481	
New York	4	5.842	0.547	
Paris	5	5.623	0.531	
Santiago	13	4.158	0.426	
Tokyo	6	5.431	0.518	
Toronto	8	5.084	0.493	
Dubai	15	3.499	0.379	<0.415
Johannesburg	14	3.889	0.407	

## 4 Discussion

### 4.1 Analysis of factors influencing the level of healthy cities

Based on the entropy weight analysis results (Figure 2), the highest weighting is assigned to the metric concerning the kilometers of bicycle paths and lanes per 100,000 population (0.068). This indicates that the said metric is a critical determinant in evaluating the healthiness of urban areas. The indicator reflects the level of a city’s bicycle infrastructure development, revealing the bicycle network’s density and demonstrating the city’s strategic investment and policy prioritization in sustainable transportation planning (39). Moreover, this metric is associated with multiple societal values, including promoting healthy lifestyles, reducing environmental pollution, enhancing economic benefits, and improving residents’ quality of life (40). The findings of Heath et al. support this study’s conclusions. Research by Heath et al. (41–45) has demonstrated that sustainable transportation infrastructure (such as cycling, walking, and public transit) can foster positive health patterns. Specifically, creating an appropriate environment and facilities for cycling in urban areas significantly impacts improving residents’ health levels. Furthermore, a study by Egiguren et al. (46) assessed the risks and benefits of mortality associated with replacing car travel with cycling (both traditional and electric bikes) among urban populations aged 20 to 64 in 17 countries. The findings suggest that achieving a high rate of bicycle use by 2050 could prevent 205,424 premature deaths annually (assuming 100% of bicycle trips replace car travel). In October 2023, the European Union announced the “European Bicycle Declaration,” which officially recognizes the significant role of cycling in decarbonizing the EU’s transport sector, further acknowledging the essential contribution of bicycle transportation to the healthy development of cities. In contrast, Signee to Milan Urban Food Policy Pact holds the least weight (0.005), indicating a relatively minor impact on the comprehensive assessment of urban health. However, the MUFPP aims to address challenges faced by the global food system, such as unhealthy diets, food security, food waste, and the environmental impacts of agriculture. The role of this pact in developing healthy cities is long-term and incremental. It positively affects urban residents’ health by improving the urban food environment and promoting healthy eating habits among residents (47). For instance, the Baltimore Healthy Eating Zones project, initiated in 2012, has encouraged healthier food choices through interventions in corner stores, leading to a reduction in overweight and obesity rates among low-income African American youth (48). This project continues to have an impact to this day. The study failed to account for potential confounding variables that could impact urban health levels, such as narrow cycling spaces, uneven road surfaces, and poor maintenance of bicycle lanes. These factors might lead to biased results and an inaccurate assessment of the benefits to residents’ health. When formulating policy recommendations, it is essential to involve experts from multiple fields to consider all potential confounding variables affecting health levels comprehensively. This approach ensures that healthy city planning and design are scientifically sound and practical in real-world applications, ultimately better promoting and improving urban health levels.

When comparing the weightings of indicators across different environmental categories (see Figure 2), it becomes clear that a distinct emphasis is placed on the impact weights assigned to secondary indicators within each environmental category. Taking the air quality category as an example, the percentage of population living within 500 m of highway or major road (0.015) plays a significant role in gauging the health level of urban air quality. This indicator, which outlines the proportion of residents living close to major traffic arteries, serves as a critical measure of residents’ convenience to transportation and holds significant importance in assessing the health risks (such as noise and air pollution) that residents may face. Moreover, this indicator also illuminates the balance struck by urban planning and development strategies between facilitating transportation convenience and ensuring the quality of residential environments (49). Human activities are identified as a primary source of poor air quality (50). Higher population densities lead to reduced urban green spaces, increased building density, traffic congestion, and inadequate urban air circulation, elevating PM2.5 concentration levels and deteriorating local air quality (51). However, research conducted by Karathodorou et al. (52) indicates that densely populated cities exhibit significantly lower *per capita* fuel consumption and car usage rates due to the availability of public transportation and shorter average commute times, reducing air pollution (53). The variation in research findings may be attributed to factors such as urban planning and infrastructure development (54), types of cities and environmental protection policies (51, 55), urban economic conditions and residents’ behavioral habits (56), as well as technological advancements, particularly innovations in the energy and transportation sectors (57). When measuring the percentage of population living within 500 m of highway or major road, it is crucial to consider the impact of confounding factors such as topography and meteorological conditions (e.g., wind speed and direction). These factors can directly influence the dispersion patterns and concentrations of pollutants, affecting air quality and ultimately impacting residents’ quality of life and health. Given these factors’ complex and dynamic interactions, future research should extensively utilize Geographic Information Systems (GIS) for evaluation, analysis, and prediction. This approach will aid in developing more effective air quality management strategies and urban planning policies.

### 4.2 Analysis of difference in environmental categories of healthy cities

Horizontal comparison of proximity values corresponding to environmental categories (Table 3) reveals significant spatial heterogeneity within the same environmental category across different cities. Taking the transport category as an example, its impact on healthy cities is relatively significant (0.239). This aligns with the findings of Nieuwenhuijsen et al. and Khreis et al. (58, 59), who have emphasized the significant influence of urban transportation planning and policies on public health, noting that motor vehicle collisions and traffic-related environmental exposures lead to premature deaths and substantial disease burdens. Although current trends are concerning, new evidence suggests that promoting healthy and sustainable transportation infrastructure, as well as adopting modes of transportation such as cycling, walking, and public transit, can effectively encourage positive travel behaviors (60, 61) and potentially reduce traffic-related environmental exposures (62), thereby improving the health status of cities. In this category, Singapore exhibits the highest proximity value (0.592), indicating that the city performs best in transportation. Singapore is internationally renowned for its integrated land use and transportation planning approach. By emphasizing public transit-led axial development, enhancing the levels and network of public transportation services, advocating a green transportation system primarily consisting of “slow travel + public transit,” and implementing the transit-oriented development (TOD) model, Singapore has established a travel mode hierarchy of “walking − cycling − Taking (public transportation)” that exemplifies ease and efficiency. These measures have effectively created Singapore’s more livable, healthy, high-quality urban living environment. In contrast, Johannesburg exhibits a poorer performance in this category (0.254), characterized by dispersed commuting and cycling spaces: a lack of integration between cycling activities and urban public transportation infrastructure, most public transportation stations are not easily accessible by non-motorized transport, and a deficiency in shared infrastructure. These issues hinder the city’s progress towards healthier development (63).

**Figure 5:**
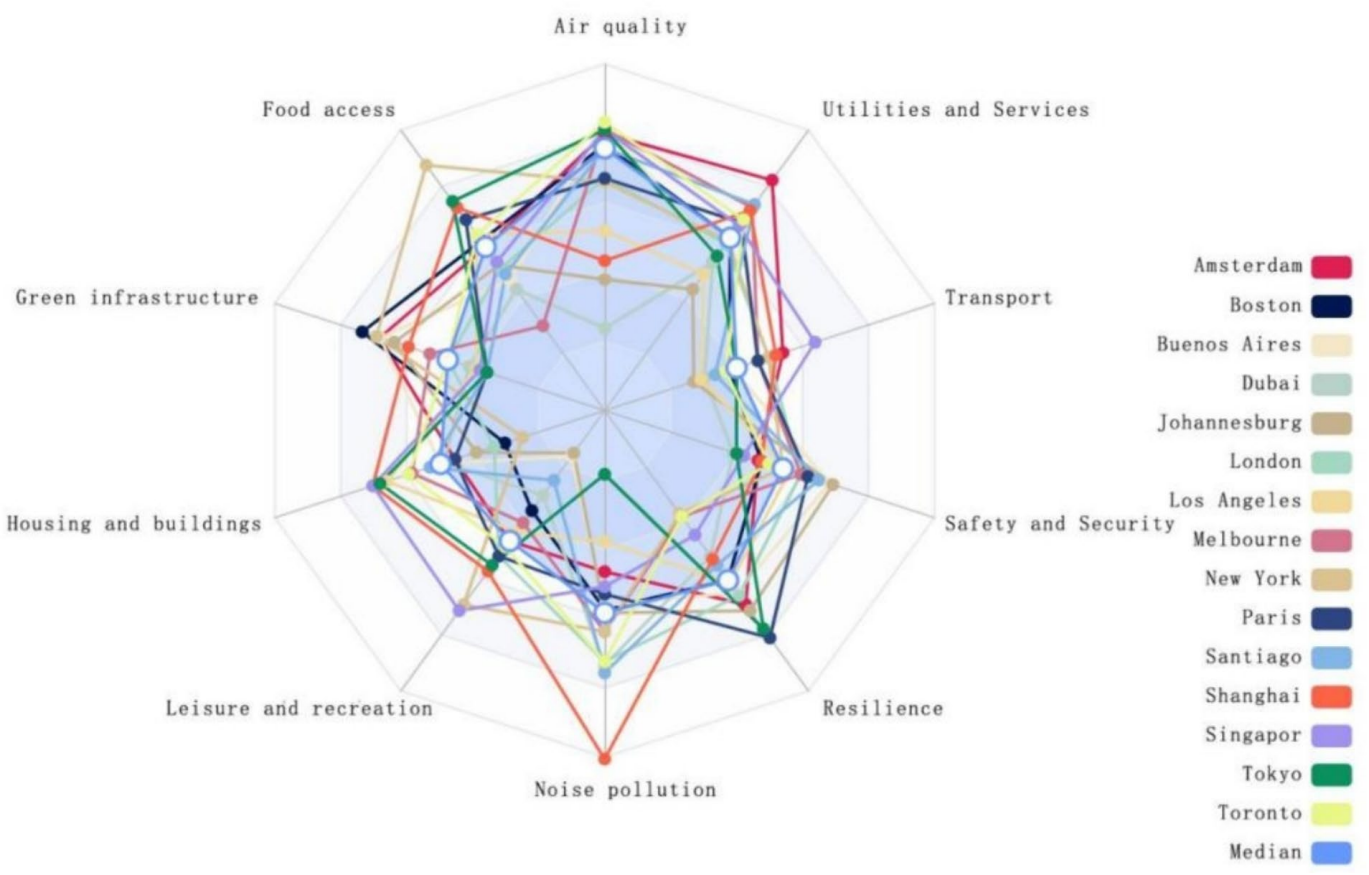
Radar chart.

A vertical comparison of the proximity values corresponding to environmental categories (Table 3) suggests that healthy cities can be categorized into two types: those with balanced development and those with unbalanced development. A city possesses balanced, healthy development if the proximity values for all 10 environmental categories are above the median. Conversely, suppose the proximity value of any single environmental category falls below the median. In that case, it indicates a significant developmental shortfall, classifying the city as having unbalanced development. To facilitate a more intuitive observation of the cities’ healthy development status, this study employs radar charts to contrast the proximity values of different environmental categories for each city against the median proximity value of each category (Figure 5). Examining 15 cities reveals a prevailing state of unbalanced development across the board. Taking Singapore and Dubai as examples, it is noted that Singapore ranks highly in environmental categories such as air quality (0.746), leisure and recreation (0.658), and housing and buildings (0.653). However, its proximity value for green infrastructure (0.352) is ranked only 12th. This may be attributed to the scarcity of land caused by its high-density, compact urban characteristics and ineffective environmental regulation (64). Despite these challenges, Singapore’s achievements in air quality, leisure and recreation, and housing construction are credited to implementing strict environmental laws and policies, urban planning oriented towards residents’ quality of life, and using sustainable design and construction technologies.

On the other hand, Dubai, while underperforming in categories such as air quality, leisure and recreation, and transport, demonstrates exemplary performance in noise pollution management. The city, by employing noise monitoring systems, precise noise index assessments, and the implementation of open data sharing and institutional collaboration, provides support for government agencies in formulating noise reduction strategies to achieve a more tranquil and healthy urban environment (65). Even cities with a higher overall health rating have shortcomings that constrain their progression towards enhanced healthfulness. Similarly, those ranked lower overall may still possess commendable practices that can serve as valuable references. Conversely, cities that rank lower overall may still possess commendable successful experiences worth learning from. Within the context of globalization, Liu et al. (66) have discovered that uneven development within cities is related to public health risks, ecosystem destruction, and social inequalities. These issues lead to unequal urban development and may widen the gap between cities. Nijman et al. (67) argue that phenomena such as economic disparities, housing inequality, food deserts, spatial mismatches, and unequal access to government services exist within urban development. These issues stem primarily from the demands of economic restructuring and quality improvement in the context of the new economic normal (27), differentiation of interests among different groups (68), uneven distribution of organic food stores (69), urban expansion and decentralization of work (70), and the limited diffusion intensity of policies (71), among other factors. Consequently, Singapore needs to improve its weaknesses further to prevent the “barrel effect” in developing healthy city construction. In contrast, Dubai urgently requires the integration of various forces to push forward comprehensively.

### 4.3 Overall analysis of healthy cities level

The comprehensive health status of urban environments results from collaborative efforts across multiple sectors. An assessment employing the integrated entropy weight-TOPSIS and RSR methods indicates that the overall development level of healthy cities is relatively low. Singapore, Shanghai, and Amsterdam rank within the top three, denoting a higher echelon within the sampled cities. In contrast, the remaining 80% of cities are deemed to have moderate to low levels of health, suggesting a global imperative for holistic urban health development. Following the promulgation of the United Nations’ 2030 Agenda for Sustainable Development in 2016, the agenda has been extensively promoted worldwide, offering a strategic blueprint for the sustainable evolution of urban areas, with a keen emphasis on reducing the disparities in health benefit distribution. Singapore’s preeminent position in the global health cities rankings can be ascribed to the foresight of its government’s long-term planning. This encompasses the creation of a highly effective health care system and a robust medical insurance framework, the application of regulatory measures grounded in the rule of law, and the holistic implementation of initiatives such as the “Garden City” vision, the “Home Ownership for Public Housing” policy, and the equalization of public services (72). As one of China’s earliest megacities to propose the construction of a healthy city, Shanghai has methodically carried out eco-environmental and health applicability and cutting-edge scientific and technological research focusing on safeguarding public health. Furthermore, environmental health risk pilot surveys and evaluations have been conducted in typical pilot areas, providing a robust basis for further enhancing health city management (73). Against the backdrop of the “Dutch National Bicycle Plan,” Amsterdam has endeavored to create a compact, high-density, pedestrian-friendly, and bicycle-friendly city to foster a bicycling-friendly environment, thereby improving the health and well-being of residents (74). By analyzing and identifying these successful cases, valuable insights and strategies can be provided for cities with lower health levels. These can be promoted and implemented globally, significantly advancing the construction and development of healthy cities worldwide. However, many city governments need more means and tools to monitor the progress of implementation measures, leading to urban sprawl, mismanagement, displacement of residents, inadequate public health infrastructure, and land expropriation, exacerbating inequality in urban areas (75). Hence, it is evident that achieving sustainable development for global healthy cities remains a significant and challenging task.

To scientifically measure the development level of healthy cities, combining the Comprehensive Healthy City Index scores with an analysis of balanced development is essential. Qing et al. (76) pointed out that a city is considered a high-quality, healthy city if it has a high level of healthy development and balanced progress among its constituent factors. Conversely, a city is categorized as a low-quality healthy city if its healthy development level is low and its constituent factors are unevenly developed. The current study shows that all 15 cities exhibit unbalanced development, falling into the low-quality healthy city development model. Therefore, in formulating relevant policies, it is crucial to fully consider the objective reality of these cities’ overall low development levels and the significant differences in environmental categories among cities. This approach allows for tailored and precise policy-making to address the challenges of unbalanced and inadequate urban development, promoting high-quality urban spaces focused on residents’ health and well-being.

### 4.4 Heterogeneity analysis of health cities evaluation indicators

To further clarify the differences among the health cities evaluation indicators, cluster analysis (K-Means) was employed to identify potential commonalities among variable indicators and to examine the heterogeneity in health indicators across different cities. The results were calculated using the mean ± standard deviation for differential analysis. As shown in Table 5, there are no significant differences among 53 indicators, including Concentration of PM2.5 μg/m^3^ (*p* = 0.288 > 0.05), availability (home): number of food stores selling fruits and vegetables within 500 m of domestic properties (*p* = 0.134 > 0.05), and hectares of green area per 100,000 population (*p* = 0.905 > 0.05). This indicates that these indicators share common characteristics in evaluating healthy cities and are suitable for widespread application across various countries or cities. Significant disparities were identified among five indicators: m2 of public outdoor recreation space *per capita* (*p* = 0.029 < 0.05), greenhouse gas emissions (*p* = 0.043 < 0.05), residential electricity use *per capita* (*p* = 0.028 < 0.05), electricity consumption of public building per year (*p* = 0.000 < 0.05), and kilometers of light passenger public transport system per 100,000 people (*p* = 0.019 < 0.05). These findings indicate substantial characteristic differences among cities concerning these indicators. Therefore, when applying these indicators for the health assessment and planning of specific cities, it is imperative to consider the actual circumstances of regional culture, economic conditions, and social systems. Implementing targeted evaluations and strategies is essential to ensure the scientific rigor and practicality of the assessment.

**Table 5:** Results of single indicator clustering analysis in the evaluation index system of healthy cities.

Indicator code	Cluster category (Mean ± SD)		F	P
	Category 1 (*n* = 13)	Category 2 (*n* = 2)		
A1	16.846 ± 12.185	7.0 ± 0.0	1.226	0.288
A2	25.231 ± 9.203	22.5 ± 0.707	0.165	0.691
A3	32.077 ± 21.55	16.5 ± 2.121	0.98	0.340
A4	66.986 ± 23.816	72.35 ± 22.698	0.089	0.771
A5	18.722 ± 7.956	16.5 ± 4.243	0.143	0.711
F1	0.923 ± 0.277	1.0 ± 0.0	0.144	0.710
F2	3.877 ± 1.702	1.865 ± 0.983	2.552	0.134
F3	52.356 ± 20.638	41.0 ± 18.385	0.533	0.478
F4	11.538 ± 4.115	15.0 ± 2.828	1.278	0.279
F5	6.077 ± 3.73	10.5 ± 6.364	2.125	0.169
F6	89.727 ± 5.208	97.065 ± 0.658	3.722	0.076^*^
G1	88.715 ± 57.141	93.88 ± 39.711	0.015	0.905
G2	9.468 ± 6.107	9.75 ± 11.95	0.003	0.957
G3	66.849 ± 27.852	92.115 ± 10.402	1.528	0.238
H1	24.1 ± 17.405	23.7 ± 19.375	0.001	0.977
H2	267.923 ± 180.282	156.5 ± 68.589	0.709	0.415
H3	43.731 ± 25.537	74.9 ± 35.497	2.409	0.145
H4	12.308 ± 1.601	13.0 ± 1.414	0.33	0.576
H5	16.819 ± 11.137	22.0 ± 4.243	0.402	0.537
H6	22.317 ± 16.09	20.36 ± 15.754	0.026	0.875
L1	1.733 ± 1.96	1.605 ± 1.464	0.008	0.932
L2	8.485 ± 5.478	18.4 ± 2.263	6.065	0.029^**^
L3	86.154 ± 41.725	96.0 ± 1.414	0.105	0.752
L4	12.846 ± 8.896	17.5 ± 9.192	0.472	0.504
L5	59.154 ± 39.61	68.0 ± 59.397	0.079	0.783
L6	7.615 ± 2.694	10.5 ± 0.707	2.141	0.167
N1	43.819 ± 9.923	41.5 ± 0.707	0.103	0.754
N2	32.577 ± 5.944	34.0 ± 2.828	0.106	0.750
N3	16.246 ± 4.395	14.25 ± 1.485	0.384	0.546
N4	21.138 ± 5.059	17.15 ± 2.616	1.142	0.305
R1	9.205 ± 4.926	17.275 ± 0.431	5.037	0.043^**^
R2	7711.308 ± 3772.307	14652.0 ± 2757.716	6.086	0.028^**^
R3	86371.769 ± 11073.124	150250.0 ± 32055.979	36.794	0.000^***^
R4	12.829 ± 5.988	22.12 ± 12.332	3.34	0.091^*^
R5	28.395 ± 16.461	15.135 ± 21.022	1.073	0.319
R6	1.227 ± 1.081	1.32 ± 0.014	0.014	0.908
R7	29.492 ± 16.011	27.9 ± 1.273	0.019	0.894
R8	26.385 ± 6.899	25.0 ± 0.0	0.076	0.788
R9	26.285 ± 5.598	30.5 ± 0.707	1.063	0.321
S1	48.939 ± 19.039	49.43 ± 5.883	0.001	0.972
S2	42.742 ± 17.89	42.43 ± 4.511	0.001	0.981
S3	51.176 ± 20.399	54.47 ± 2.418	0.049	0.828
T1	60.844 ± 19.435	41.6 ± 13.294	1.772	0.206
T2	106.49 ± 150.291	40.6 ± 36.911	0.359	0.559
T3	218.162 ± 208.7	228.35 ± 214.748	0.004	0.950
T4	163.303 ± 95.147	352.5 ± 65.761	7.141	0.019^**^
T5	6370.462 ± 5741.936	7264.0 ± 4143.646	0.044	0.838
T6	59.162 ± 9.379	47.5 ± 6.364	2.796	0.118
T7	14.656 ± 7.17	9.565 ± 0.233	0.947	0.348
T8	86.492 ± 5.15	83.58 ± 10.861	0.438	0.520
T9	95.154 ± 85.385	117.5 ± 0.707	0.129	0.726
T10	60.846 ± 27.88	97.5 ± 27.577	3.001	0.107
T11	16.077 ± 7.566	17.5 ± 7.778	0.061	0.809
U1	91.147 ± 18.54	99.595 ± 0.474	0.39	0.543
U2	82.923 ± 36.762	100.0 ± 0.0	0.405	0.535
U3	83.474 ± 15.38	80.55 ± 7.283	0.067	0.800
U4	15.531 ± 6.992	11.45 ± 3.465	0.627	0.443
U5	48.146 ± 16.685	39.4 ± 5.091	0.512	0.487

***, **, and *, respectively, represent significance levels of 0.01, 0.05, and 0.1.

**Figure 6:**
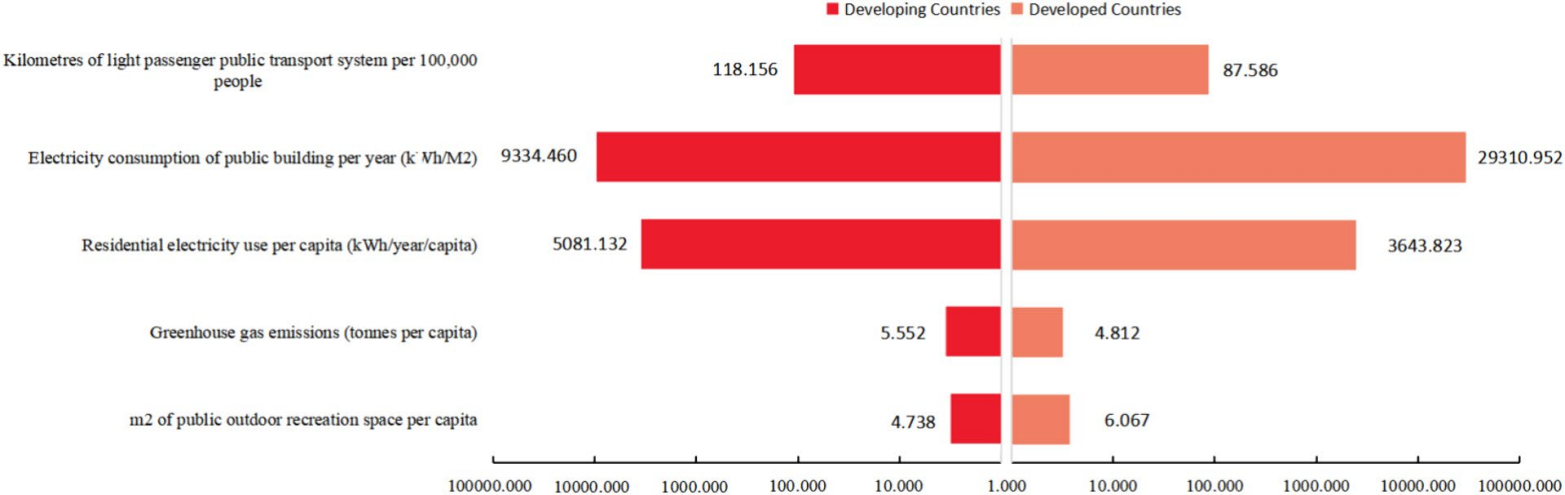
Comparison of standard deviations of individual indicators between developing and developed countries.

This investigation categorizes 15 cities into developing and developed nations, computing the standard deviation across five distinct indicators to evaluate the dispersion of data. An elevated standard deviation signifies increased dispersion, indicating pronounced disparities among cities within each classification; conversely, a lower standard deviation suggests minimal differences. Figure 6 illustrates that developed nations demonstrate significant standard deviation concerning two metrics: m2 of public outdoor recreation space *per capita* and electricity consumption of public buildings per year. These disparities underscore the presence of substantial variation between cities, likely attributable to variations in municipal policy decisions, funding allocation priorities, and disparities in the lifestyles and cultural norms of the inhabitants. M2 of public outdoor recreation space *per capita* is defined as the average amount of outdoor space accessible to each individual, which is intrinsically linked to the residents’ living quality and health. An increased *per capita* provision of such spaces affords residents more significant opportunities for physical exercise, nature engagement, and community involvement, thereby enhancing life quality and fostering physical well-being. Moreover, it alleviates the psychological effects of congested environments prevalent in densely populated urban areas (77). Electricity consumption of public buildings per year quantifies the total energy utilization within a given period by a range of public infrastructures, including but not limited to government bodies and educational, medical, and cultural institutions. This indicator sheds light on the efficiency of operations and energy usage of public service buildings. Structures boasting higher energy efficiency manage to reduce electricity consumption while maintaining operational capabilities, reducing greenhouse gas emissions and environmental pollution (78). In the progression toward health-centric urban development, governments play a crucial role in improving urban air quality and enhancing residents’ health by implementing strategies to reduce pollution and lower energy consumption.

In developing countries, cities exhibited a higher degree of dispersion across three metrics: greenhouse gas emissions, residential electricity use *per capita*, and electricity consumption of public buildings per year. Such disparities likely mirror the variations in the degree of industrialization, the configuration and efficiency of energy use, and the levels of investment in infrastructure. Greenhouse gas emissions quantify the average emissions discharged over a specified timeframe by a nation or region, divided by its total population (79). Employed to evaluate the average contribution of residents from various countries or areas to climate change, this indicator measures their collective impact on global climate challenges. Effective management of greenhouse gas emissions is crucial for diminishing the negative consequences associated with global warming, preserving the ecological systems of urban areas, and ensuring the health of inhabitants and the sustainable development of cities over the long term. Residential electricity use *per capita* is determined by dividing the total electricity consumed by the residential sector of a country or region over a specified period by its overall population (80). This indicator gauges the average electricity consumption per resident, providing an essential measure for assessing residents’ quality of life, economic development, and the efficiency of electricity usage. It plays a pivotal role in monitoring the allocation of electrical resources, tracking alterations in consumption behaviors, and underpinning relevant energy policies and strategic planning. Through the optimization of *per capita* electricity consumption, it is feasible to fulfill the everyday needs of the populace while mitigating environmental strains, thereby fostering the sustainable progression of cities. Kilometers of light passenger public transport system per 100,000 people individuals evaluates the urban public transportation network’s density and accessibility (81). It offers insights into the coverage provided by public transport, the efficacy of urban planning, and governmental endorsement of eco-friendly commuting practices. An extensive network of public transit routes promotes public transportation among residents, which helps ease traffic congestion, decrease greenhouse gas emissions from private car usage, and improve travel efficiency and convenience for urban dwellers. This, in turn, supports the sustainable advancement of healthy city environments.

Therefore, it is crucial to balance standard and individualized metrics in formulating and applying global health city assessment indicators. This balance ensures that the assessment of healthy cities retains universality and accounts for the specific needs and contexts of different locales. This approach enables a more scientific assessment of urban health development status and assists in formulating urban health policies and planning.

### 4.5 Recommendations

In light of this, reflecting on the successful experiences of leading global cities in health development and aiming to improve evaluation practices, it is advisable to proceed from four aspects: government leadership, regulatory planning, consensus building, and strategic implementation. The following recommendations are proposed to foster the sustainable development of urban health on a global scale.

#### 4.5.1 Strengthening the policy support system

The advancement of a robust policy framework for healthy urban planning is posited as a prerequisite for the development of health-oriented urban spaces, ensuring effective construction and management, optimized mechanisms, and accessible information to safeguard the evolution of healthy cities. Drawing from Singapore’s developmental paradigm, it is proposed that, at the legislative level, policies pertinent to public health be crafted and refined, with the enactment of corresponding laws and regulations, thus providing a legal basis for regulatory governance. On a mechanistic level, the refinement of decision-making processes is imperative, necessitating a tight integration of evaluation outcomes and strategic adjustments, bolstering demonstration and incentivization efforts, and enhancing the safety and health emergency command and support system. At the implementation level, drawing upon the advanced experiences of Shanghai, the establishment of a periodic and continuous evaluation system for urban health initiatives proves beneficial in understanding the efficacy levels post-policy implementation. Moreover, the enhancement of international exchanges and collaboration provides access to global best practices and avant-garde experiences in health cities construction, thereby presenting new developmental opportunities for the optimization and reinforcement of healthy urban environments.

#### 4.5.2 Refining urban spatial planning

Establishing a healthy, safe, and resilient spatial structure is a critical foundation for executing urban health development strategies. Therefore, it is imperative to integrate the concept of health across the entire lifecycle into the current national spatial planning. This involves identifying significant ecologically sensitive areas within cities and regions that substantially impact citizens’ health, proposing appropriate health graduation standards, and optimizing the allocation of health-related spatial resources. Following the example set by Dubai, in the new context of accelerated data openness and sharing, it is vital to fully leverage the internet and data-sharing platforms to integrate urban and population health network data. This approach dismantles barriers across public health, medical insurance, urban–rural planning, ecological environment, and safety disaster prevention sectors. It provides functions for the release, inquiry, service, and emergency alert of ample data information pertinent to healthy urban spaces, thereby ensuring the spatial implementation of planning and construction schemes.

#### 4.5.3 Expanding paths for multi-stakeholder participation

The government’s leadership and interdepartmental collaboration are significant driving forces in advancing healthy urban construction. Drawing from Amsterdam’s successful experiences in social mobilization and citizen participation, it is advocated to diversify the institutions implementing evaluations. This promotes a shift from government-led initiatives to societal collaboration, thereby stimulating citizen engagement in the construction of healthy cities and the active allocation of health resources. It is essential to establish and refine investment platforms dedicated to this purpose to facilitate the construction of healthy cities. Moving away from paths that rely solely on government investment can encourage market-oriented approaches and promote participation from private capital. This strategy helps to mitigate the risk of ‘involution’—a term that describes inward, competitive intensity without corresponding increases in value—in the design and implementation of related public policies. By guiding healthy cities’ development toward a collaborative governance model, a win-win situation can be achieved that simultaneously benefits economic, social, and ecological interests.

#### 4.5.4 Incorporating distinctive health indicators

The construction of healthy cities in different cities should align with their unique health conditions, economic development levels, and social backgrounds. To ensure the accuracy of evaluations, differentiated assessments should be conducted by optimizing the evaluation indicator system for healthy cities. This involves considering various aspects such as the development stage, geographical location, and cultural background of different types of cities. As the development of healthy cities progresses, in addition to universal indicators, it is necessary to introduce indicators that reflect the characteristics of each city and develop evaluation methods that balance continuity and localization. For cities in developing countries, evaluation indicators should focus on infrastructure and technologies that reflect urban development and support sustainable lifestyles rather than merely meeting residents’ basic needs. In contrast, for cities in developed countries, there should be a greater emphasis on technological and industrial innovation and indicators that reflect climate change. By establishing governance models for healthy cities tailored to their characteristics, scientific support can be provided for the fine-tuned construction and management of healthy cities. This will help enhance the convergence of local initiatives with global standards in health city development.

## 5 Conclusion

Drawing upon the Healthy City concept proposed by the World Health Organization, this study employs the BRE international set of indicators for evaluating healthy cities. Using the entropy weight-TOPSIS method and the rank sum ratio (RSR) technique, a comparative assessment and hierarchical classification of the health development statuses of 15 cities globally is conducted. Cluster analysis is deployed to discern common and unique indicators, allowing for necessary adjustments in the health evaluation of different cities based on their developmental characteristics. This provides a foundation for formulating specific recommendations for improvement, aiming to serve as a reference for constructing healthy cities. The ultimate goal is to reduce developmental disparities between cities and to promote the balanced and sustainable development of healthy cities worldwide. The primary outcomes of the research are as follows:

1. Sub-indicator Importance: In assessing urban health, the kilometers of bicycle paths and lanes per 100,000 population emerges as the paramount indicator. Conversely, the effect of signee to Milan Urban Food Policy Pact has been minimal. The emphasis varies across different environmental categories; for instance, the percentage of the population within 500 meters of a highway or major road significantly influences the air quality category, while the *per capita* green space has a more considerable impact on the green infrastructure category.
2. Environmental Categories Importance: From the perspective of various environmental categories, the transport category has the most significant impact on assessing healthy cities. In contrast, the safety and security category exhibits the most minor influence. Specific disparities remain among different cities and environmental categories, indicating imbalanced health development. Using Singapore and Dubai as examples, Singapore shows commendable performance in air quality, leisure and recreation, and housing and buildings. However, its performance in the green infrastructure category is merely moderate. On the other hand, Dubai displays subpar performance in air quality, leisure and recreation, and transport categories yet ranks favorably in the noise pollution category.
3. Composite Rankings of Healthy Cities: The overall level of healthy city development in the 15 cities is relatively low, and all fall into the low-quality healthy city development model. Overall, cities like Singapore, Shanghai, and Amsterdam have achieved a higher level of health infrastructure development. Others, including Boston, Buenos Aires, London, Los Angeles, Melbourne, New York, Paris, Santiago, Tokyo, and Toronto, fall into the medium range of the spectrum. Meanwhile, Dubai and Johannesburg are ranked lower regarding their health infrastructure development.
4. Common and Unique Indicators: Concentration of PM2.5 μg/m^3^, availability (home): number of food stores selling fruits and vegetables within 500 m of domestic properties., and hectares of green area per 100,000 population, among other 53 indicators, constitute the common indicators of the health cities evaluation system, demonstrating universality. However, for different cities, it is essential to highlight distinctive indicators such as m2 of public outdoor recreation space *per capita*, greenhouse gas emissions, residential electricity use *per capita*, electricity consumption of public buildings per year, and kilometers of light passenger public transport system per 100,000 people. These five indicators aim to reflect the health status of cities more accurately.

In the global assessment of healthy cities, individual indicators, environmental categories, and overall health city rankings are designed to diagnose the weaknesses cities face in their development process effectively. Specifically, ranking by individual indicators helps to establish strategic priorities, guiding the specific areas of governance that need prioritized development and improvement across various environmental categories. Ranking by environmental categories helps fully reflect the health disparities among different evaluation subjects, identifying a city’s strengths and issues and optimizing resource allocation to ensure the balanced development of healthy cities. The global comprehensive ranking of healthy cities helps to assess the overall health status of cities, facilitating the discovery of exemplary practices that can provide reference and inspiration for cities with lower health levels, promoting international learning and experience exchange, and accelerating the construction of global healthy cities toward sustainable and equitable urban development goals. Generic indicators aim to provide reliable support for formulating universally applicable policies for healthy city development, ensuring their broad applicability and operability. Distinctive indicators reflect the unique health needs of each city. By identifying these indicators, targeted health interventions can be implemented to meet the specific health needs of different cities, driving high-quality development of healthy cities.

Selecting Alpha-level cities, which are highly connected in the global economic network, as the primary sample for assessing the development level of healthy cities is straightforward and widely accepted. However, with rapid economic growth, improving objective well-being has not significantly led to a corresponding increase in public health and subjective happiness (82). Therefore, future research should avoid the limitations of a single economic perspective and consider economic, political, cultural, social, and ecological civilization aspects to expand the research dimensions, leading to more accurate and comprehensive conclusions. Additionally, in terms of research methods, although the entropy method has the advantage of objective weighting, it does not consider the decision-makers subjective preferences and importance judgments when calculating weights based on the objective distribution of indicator data. It also assumes that each indicator is independent without considering possible significant correlations between them. This method of ignoring decision-makers preferences and inter-indicator correlations may lead to unreasonable allocation of indicator weights, resulting in biased assessment results and affecting the accuracy of overall healthy city scores. In subsequent research, it is necessary to comprehensively consider the decision-makers’ subjective judgments and inter-indicator correlations, introducing subjective weights of decision-makers, and combining methods such as the entropy method, CRITIC method, and AHP method to compare results from different methods and identify and adjust potential biases.

## Data availability statement

The original contributions presented in the study are included in the article/supplementary material, further inquiries can be directed to the corresponding author.

## Author contributions

YW: Supervision, Writing – review & editing. YL: Data curation, Investigation, Methodology, Software, Writing – original draft, Writing – review & editing. YZ: Resources, Writing – review & editing. BL: Data curation, Writing – review & editing.

## References

[1] Zhanyun W Jingjing S. International practice and trend of healthy City. Urban Insight. (2017) 6:138–48.

[2] Alirol E Getaz L Stoll B Chappuis F Loutan L. Urbanisation and infectious diseases in a globalised world. Lancet Infect Dis. (2011) 11:131–41. doi: 10.1016/S1473-3099(10)70223-1, PMID: 21272793 PMC7106397

[3] Maoyang Y. Current situation and hotspots analysis of healthy City researches in China. Chin J Health Educ. (2019) 35:898–902. doi: 10.16168/j.cnki.issn.1002-9982.2019.10.008

[4] Lin M Liang D Ying Z. Development and reflection on healthy cities in China. Med Philos. (2017) 38:5–8.

[5] Pan J. Jingjing S. Blue book of cities in China:annual report on URBAN DEVELOPMENT of China no.9. Beijing: Social Sciences Academic Press, (2016): 1–28.

[6] World Health Organization. Ottawa charter for health promotion, 1986. Ottawa: World Health Organization, Regional Office for Europe (1986).

[7] Xiji J Dan Y Lan W. The evolution of global healthy City movement and the function of urban planning. Urban Planning Int. (2020) 35:128–34. doi: 10.19830/j.upi.2019.585

[8] Shaohua T Qixiao H Chun Y. Active planning intervention techniques for healthy cities: Ascale conversion effect. Sci Technol Rev. (2020) 38:34–42.

[9] Lan W Yinghui J Wenyao S Fangfang J. Quantitative health impact assessment for urban planning. Planners. (2021) 37:72–7.

[10] Capolongo S Rebecchi A Dettori M Appolloni L Azara A Buffoli M. Healthy design and urban planning strategies, actions, and policy to achieve salutogenic cities. Int J Environ Res Public Health. (2018) 15:2698. doi: 10.3390/ijerph15122698, PMID: 30501119 PMC6313765

[11] Lei S. Healthy City: theoretical characteristics and future actions. J Environ Prot. (2020) 4:50–8. doi: 10.16619/j.cnki.rmltxsqy.2020.04.005

[12] Guangyou L Xi L Aiqiong J Jian C. Discussion on the construction of index system indicators for evaluation of healthy cities. Smart Healthcare. (2018) 4:3–4.

[13] Dong L Song H Fengjing L. Review and prospects of the healthy City evaluation systems. J Hum Settle West China. (2023) 38:17–23. doi: 10.13791/j.cnki.hsfwest.20230203

[14] World Health Organization (WHO). Healthy cities: Guide note for the healthy cities indicators. Copenhagen: MCAP indicators, WHO Regional Office for Europe (1992).

[15] World Health Organization (WHO). WHO healthy cities—revised baseline healthy cities indicators. Copenhagen: WHO Regional Office for Europe (1998).

[16] Phillis YA Kouikoglou VS Verdugo C. Urban sustainability assessment and ranking of cities. Comput Environ Urban Syst. (2017) 64:254–65. doi: 10.1016/j.compenvurbsys.2017.03.002

[17] Van den Bosch M Sang ÅO. Urban natural environments as nature-based solutions for improved public health–a systematic review of reviews. Environ Res. (2017) 158:373–84. doi: 10.1016/j.envres.2017.05.040, PMID: 28686952

[18] Zhou N He G Williams C Fridley D. ELITE cities: a low-carbon eco-city evaluation tool for China. Ecol Indic. (2015) 48:448–56. doi: 10.1016/j.ecolind.2014.09.018

[19] Zhang X Bayulken B Skitmore M Lu W Huisingh D. Sustainable urban transformations towards smarter, healthier cities: theories, agendas and pathways. J Clean Prod. (2018) 173:1–10. doi: 10.1016/j.jclepro.2017.10.345

[20] World Health Organization. 2018 global reference list of 100 core health indicators (plus health-related SDGs). Geneva: World Health Organization (2018).

[21] National Patriotic Health Campaign Committee. Notice of the National Patriotic Health Campaign Committee on the issuance of the National Healthy City Evaluation Index System (2018) [EB/OL]. (2018-03-28) [2022-03-31]. Available at: http://www.nhc.gov.cn/jkj/s5899/201804/fd8c6a7ef3bd41aa9c24e978f5c12db4.shtml.

[22] Remington PL Catlin BB Gennuso KP. The county health rankings: rationale and methods. Popul Health Metrics. (2015) 13:1–12. doi: 10.1186/s12963-015-0044-2 PMC4415342 25931988

[23] Barboza EP Cirach M Khomenko S Iungman T Mueller N Barrera-Gómez J. Green space and mortality in European cities: a health impact assessment study. Lancet Planet Health. (2021) 5:e718–30. doi: 10.1016/S2542-5196(21)00229-1, PMID: 34627476

[24] Lv Z Guo H Zhang L Liang D. Comparative study on the evaluation of healthy City construction in typical Chinese cities based on statistical data and land use data. Sustain For. (2022) 14:2519. doi: 10.3390/su14052519

[25] Yan D Wu S Zhou S Li F Wang Y. Healthy city development for Chinese cities under dramatic imbalance: evidence from 258 cities. Sustain Cities Soc. (2021) 74:103157. doi: 10.1016/j.scs.2021.103157

[26] Pineo H Zimmermann N Cosgrave E Aldridge RW Acuto M Rutter H. Promoting a healthy cities agenda through indicators: development of a global urban environment and health index. Cities Health. (2018) 2:27–45. doi: 10.1080/23748834.2018.1429180

[27] Haijun C Meng W. Healthy City construction in Liaoning Province, 2019: an entropy weight based TOPSIS evaluation. Chin J Public Health. (2022) 38:1214–8.

[28] Donald SH Gammack JG. Tourism and the branded city: Film and identity on the Pacific rim. London: Routledge (2016).

[29] Jiamei X Xiuhua M Xiaodan L Weiguang Y. Current situation and forecast analysis of resource allocation of general practitioners in Guangdong-Hong Kong-Macau Greater Bay Area, 2017-2021. Modern Prevent Med. (2023) 50:2037–41. doi: 10.20043/j.cnki.MPM.202212172

[30] Jinyan W Sun Shide X Dianting MW. Healthy City spatial organization framework and its promotion strategy from the perspective of health economics regional. Econ Rev. (2023) 5:79–86. doi: 10.14017/j.cnki.2095-5766.2023.0072

[31] Binglian L Ziming G. Connotation, mechanism, and path of Chinese new urbanization from the perspective of urban agglomeration spatial structure. J Xi'an Jiaotong Univ. (2023) 43:11–22. doi: 10.15896/j.xjtuskxb.202304002

[32] Junhan G Kai L Deng Yuefei Y Chenghua A. Geological suitability evaluation of underground space resources based on the entropy weight optimization method. Geol Bull China. (2023) 42:385–96.

[33] Li Z Luo Z Wang Y Fan G Zhang J. Suitability evaluation system for the shallow geothermal energy implementation in region by entropy weight method and TOPSIS method. Renew Energy. (2022) 184:564–76. doi: 10.1016/j.renene.2021.11.112

[34] Yanlei G Zhengyan Z Zhigang W. Food security evaluation based on entropy weight TOPSIS method: cutting from major grain producing area. J Agro-Forestry Econ Manage. (2019) 18:135–42. doi: 10.16195/j.cnki.cn36-1328/f.2019.02.16

[35] Chunyang L Feng W Qingyuan Y Linlin C Huiming Z. An evaluation of urban land use performance based on the improved TOPSIS method and diagnosis of its Obstale indicators: a case study of Chongqing. Resourc Sci. (2011) 33:535–41.

[36] Xunping L Qiu R Yong L. Evaluation of regional land use performance based on entropy weight TOPSIS model and diagnosis of its Obstale factors. Transactions of the Chinese society of. Agric Eng. (2016) 32:243–53.

[37] Fengdiao T. Rank sum ratio (RSR) method and its applications. J Chin Phys. (2002) 2:115–9.

[38] Fengdiao T. The problem of grading in the RSR method. Chinese journal of health. Statistics. (1993) 2:26–8.

[39] Meesit R Puntoomjinda S Chaturabong P Sontikul S Arunnapa S. Factors affecting travel behaviour change towards active mobility: a case study in a thai university. Sustain For. (2023) 15:11393. doi: 10.3390/su151411393

[40] Pucher J Buehler R. Making cycling irresistible: lessons from the Netherlands. Denmark Germany Transport Rev. (2008) 28:495–528. doi: 10.1080/01441640701806612

[41] Heath GW Brownson RC Kruger J Miles R Powell KE Ramsey LT. The effectiveness of urban design and land use and transport policies and practices to increase physical activity: a systematic review. J Phys Act Health. (2006) 3:S55–76. doi: 10.1123/jpah.3.s1.s55, PMID: 28834525

[42] Van Schalkwyk MCI Mindell JS. Current issues in the impacts of transport on health. Br Med Bull. (2018) 125:67–77. doi: 10.1093/bmb/ldx048 29309529

[43] Mueller N Rojas-Rueda D Khreis H Cirach M Andrés D Ballester J. Changing the urban design of cities for health: the superblock model. Environ Int. (2020) 134:105132. doi: 10.1016/j.envint.2019.105132.Epub, PMID: 31515043

[44] Nieuwenhuijsen MJ. Urban and transport planning pathways to carbon neutral, liveable and healthy cities; a review of the current evidence. Environ Int. (2020) 140:105661. doi: 10.1016/j.envint.2020.105661 32307209

[45] Panter J Heinen E Mackett R Ogilvie D. Impact of new transport infrastructure on walking, cycling, and physical activity. Am J Prev Med. (2016) 50:e45–53. doi: 10.1016/j.amepre.2015.09.021, PMID: 26585051 PMC4712020

[46] Egiguren J Nieuwenhuijsen MJ Rojas-Rueda D. Premature mortality of 2050 high bike use scenarios in 17 countries. Environ Health Perspect. (2021) 129:127002. doi: 10.1289/EHP9073, PMID: 34851171 PMC8634902

[47] Kim G. Portraying the urban food environment of the City of Toronto before and during the COVID-19 Pandemic through Yelp Reviews [Thesis] (2022).

[48] Shin A Surkan PJ Coutinho AJ Suratkar SR Campbell RK Rowan M. Impact of Baltimore healthy eating zones: an environmental intervention to improve diet among African American youth. Health Educ Behav. (2015) 42:97S–105S. doi: 10.1177/1090198115571362, PMID: 25829124

[49] Baghestani A Tayarani M Allahviranloo M Nadafianshahamabadi R Kucheva Y Reza Mamdoohi A. New York City cordon pricing and its’ impacts on disparity, transit accessibility, air quality, and health. Case studies on. Transp Policy. (2022) 10:485–99. doi: 10.1016/j.cstp.2022.01.009

[50] Borck R Schrauth P. Population density and urban air quality. Reg Sci Urban Econ. (2021) 86:103596. doi: 10.1016/j.regsciurbeco.2020.103596

[51] Hongwei D Ying H. The spatial effects of Beijing-Tianjin-Hebei Region’s Mog pollution, industrial structure and urbanization. Econ Theory Bus Manage. (2019) 5:4–19.

[52] Karathodorou N Graham DJ Noland RB. Estimating the effect of urban density on fuel demand. Energy Econ. (2010) 32:86–92. doi: 10.1016/j.eneco.2009.05.005

[53] Borck R. Public transport and urban pollution. Reg Sci Urban Econ. (2019) 77:356–66. doi: 10.1016/j.regsciurbeco.2019.06.005

[54] Guoxing Z Junna W Weichun L Ruikun M. Does urban agglomeration construction improve or deteriorate urban air quality? — an empirical test based on the difference-in-differences model. Syst Engineer Theory Pract. (2022) 42:1245–59.

[55] Tao Z Di W Fan L. The effect and mechanism of multi-scales forms on air quality. Geogr Res. (2022) 41:1883–97.

[56] Zhou C Chen J Wang S. Examining the effects of socioeconomic development on fine particulate matter (PM2. 5) in China's cities using spatial regression and the geographical detector technique. Sci Total Environ. (2018) 20:436–45. doi: 10.3390/ijerph20042814, PMID: 29156264

[57] Zhao J Xi XI Na QI Wang S Kadry SN Kumar PM. The technological innovation of hybrid and plug-in electric vehicles for environment carbon pollution control. Environ Impact Assess Rev. (2021) 86:106506. doi: 10.1016/j.eiar.2020.106506

[58] Nieuwenhuijsen M Khreis H. Urban and transport planning, environment and health In: *Integrating Human Health into Urban and Transport Planning: A Framework* Springer Cham: Springer International Publishing AG, part of Springer Nature 2019. (2019). 3–16.

[59] Khreis H Kelly C Tate J Parslow R Lucas K Nieuwenhuijsen M. Exposure to traffic-related air pollution and risk of development of childhood asthma: a systematic review and meta-analysis. Environ Int. (2017) 100:1–31. doi: 10.1016/j.envint.2016.11.012, PMID: 27881237

[60] Khreis H Warsow KM Verlinghieri E Guzman A Pellecuer L Ferreira A. The health impacts of traffic-related exposures in urban areas: understanding real effects, underlying driving forces and co-producing future directions. J Transp Health. (2016) 3:249–67. doi: 10.1016/j.jth.2016.07.002

[61] Heinen E Panter J Mackett R Ogilvie D. Changes in mode of travel to work: a natural experimental study of new transport infrastructure. Int J Behav Nutr Phys Act. (2015) 12:1, 81–10. doi: 10.1186/s12966-015-0239-8, PMID: 26091806 PMC4496849

[62] Geels FW. A socio-technical analysis of low-carbon transitions: introducing the multi-level perspective into transport studies. J Transp Geogr. (2012) 24:471–82. doi: 10.1016/j.jtrangeo.2012.01.021

[63] Risimati B Gumbo T Chakwizira J. Spatial integration of non-motorized transport and urban public transport infrastructure: a case of Johannesburg. Sustain For. (2021) 13:11461. doi: 10.3390/su132011461

[64] Law A Carrasco LR Richards DR Shaikh SFEA Tan CLY Nghiem LTP. Leave no one behind: a case of ecosystem service supply equity in Singapore. Ambio. (2022) 51:2118–36. doi: 10.1007/s13280-022-01735-x, PMID: 35507247 PMC9378807

[65] Bite P Z Sillo S Varfi L. Long-term noise monitoring and urban planning-a case study//INTER-NOISE and NOISE-CON congress and conference proceedings. Institute of Noise Control Engineering, (2017), 255: 5401–5406.

[66] Liu Y Du J Wang Y Xiaoyong C Jichang D Pan G. Overlooked uneven progress across sustainable development goals at the global scale: challenges and opportunities. The. Innovations. (2024) 5:100573. doi: 10.1016/j.xinn.2024.100573 PMC10876912 38379792

[67] Nijman J Wei YD. Urban inequalities in the 21st century economy. Appl Geogr. (2020) 117:102188. doi: 10.1016/j.apgeog.2020.102188 32287517 PMC7124478

[68] Hao Y Changchun F. How do housing disparity affect young People's access toPrimary jobs?—empirical study based on CFPS data. China Youth Study. (2023) 8:14–22. doi: 10.19633/j.cnki.11-2579/d.2023.0114

[69] Zhang L Wei YD. Spatial inequality and dynamics of foreign hypermarket retailers in China. Geogr Res. (2017) 55:395–411. doi: 10.1111/1745-5871.12235

[70] Wei YD Ewing R. Urban expansion, sprawl and inequality. Landsc Urban Plan. (2018) 177:259–65. doi: 10.1016/j.landurbplan.2018.05.021

[71] Guowu H. Study on the development of healthy cities in China under the background of healthy China. J Northwest Univ. (2018) 48:74–82. doi: 10.16152/j.cnki.xdxbsk.2018-03-009

[72] Jianwu X. Research on the dilemma and way out of health governance in megacities under the high risk background. Shanghai Urban Management. (2020) 29:11–9.

[73] Changmin J. Comprehensive advancement of the construction of beautiful Shanghai to help create a model of a healthy City. Health China Observation. (2023) 10:66–8.

[74] Yuejia X Hao F Li Y Yitong C Pinghao L. Circuit construction of cycling friendly cities in the background of double carbon: introduction to Pan-european master plan for cycling promotion. Urban Dev Stud. (2023) 30:1–11.

[75] Adlakha D John F. The future is urban: integrated planning policies can enable healthy and sustainable cities. Lancet Glob Health. (2022) 10:e790–1. doi: 10.1016/S2214-109X(22)00211-X, PMID: 35561712

[76] Jing Q Xiaomin S. Standard measurement and comprehensive evaluation of good life based on entropy weight TOPSIS method. Stud Ethics. (2023) 5:121–32. doi: 10.15995/j.cnki.llxyj.2023.05.001

[77] Wang H Dai X Wu J Wu X Nie X. Influence of urban green open space on residents’ physical activity in China. BMC Public Health. (2019) 19:1–12. doi: 10.1186/s12889-019-7416-7 31409316 PMC6693084

[78] Spiru P Simona PL. A review on interactions between energy performance of the buildings, outdoor air pollution and the indoor air quality. Energy Procedia. (2017) 128:179–86. doi: 10.1016/j.egypro.2017.09.039

[79] Liu D Guo X Xiao B. What causes growth of global greenhouse gas emissions? Evidence from 40 countries. Sci Total Environ. (2019) 661:750–66. doi: 10.1016/j.scitotenv.2019.01.197, PMID: 30685733

[80] Almasri RA Alshitawi MS. Electricity consumption indicators and energy efficiency in residential buildings in GCC countries: extensive review. Energ Buildings. (2022) 255:111664. doi: 10.1016/j.enbuild.2021.111664

[81] Rodríguez-Núñez E García-Palomares JC. Measuring the vulnerability of public transport networks. J Transp Geogr. (2014) 35:50–63. doi: 10.1016/j.jtrangeo.2014.01.008

[82] Shihai Z Daozhuang S. Sense of gain, happiness, and security: the era of putting people at the center. Governance. (2017) 44:41–4. doi: 10.16619/j.cnki.cn10-1264/d.2017.44.004

